# Klotho在肺癌中的研究进展

**DOI:** 10.3779/j.issn.1009-3419.2023.101.19

**Published:** 2023-06-20

**Authors:** Jing WANG, Lili ZENG, Lingping KONG, Linlin ZHANG, Jun CHEN, Diansheng ZHONG, Yaguang FAN

**Affiliations:** ^1^300052 天津，天津医科大学总医院，天津市肺癌研究所，天津市肺癌转移和肿瘤微环境重点实验室; ^1^Tianjin Key Laboratory of Lung Cancer Metastasis and Tumor Microenvironment, Tianjin Lung Cancer Institute; ^2^天津医科大学总医院肿瘤内科, Tianjin 300052, China; ^2^Department of Medical Oncology, Tianjin 300052, China; ^3^天津医科大学总医院肺部肿瘤外科, Tianjin 300052, China; ^3^Department of Lung Cancer Surgery, Tianjin Medical University General Hospital, Tianjin 300052, China

**Keywords:** 肺肿瘤, Klotho, 发病机制, 治疗, Lung neoplasms, Klotho, Pathogenesis, Treatment

## Abstract

Klotho基因最早作为一种抗衰老基因被发现，其编码的Klotho蛋白在人类的多种组织中都有表达，该蛋白最显著的功能是调节磷酸盐稳态。Klotho蛋白有抑制相关信号通路、降低氧化应激并抑制炎症反应的作用，而这些过程与癌症发生密切相关。Klotho蛋白是多种恶性肿瘤的抑制因子，其表达与肿瘤的发生发展和患者预后都有关系。肺癌是最常见的恶性肿瘤之一，其发病率和死亡率在所有恶性肿瘤中位居前列。本文就Klotho与肺癌的发病机制、治疗预后关系的研究进展进行综述，对探索与肺癌的诊疗预后相关的新标志物和新靶点有重要意义。

## Abstract (en)

Klotho gene was originally discovered as an anti-aging gene, Klotho protein encoded by Klotho gene is expressed in multiple human tissues, and its most prominent function is the regulation of phosphate homeostasis. Klotho protein possesses various activities, including inhibition of multiple signaling pathways, reducing oxidative stress and suppressing inflammation, and these activities are associated with cancer. Klotho protein is discovered as a universal tumor suppressor, and its expression is associated with tumorigenesis and prognosis of patients. Lung cancer is the most common malignancy tumor, and it is the leading cause of cancer deaths worldwide because of its high incidence and mortality. This article summarizes the research progress of the role of Klotho on pathogenesis, therapeutic effect and prognosis in lung cancer, in order to provide new biomarker and target for diagnosis, treatment and prognosis of lung cancer.

2020年全球有220万新诊断癌症病例和180万因癌症死亡病例，其中肺癌在所有癌症中的发病率位列第二，其死亡率位居第一。现今，肺癌已占新确诊癌症的1/10，占因癌症死亡人数的1/5^[1]^。目前对于肺癌的治疗已经取得了很大的进步，但部分患者的预后仍不理想。因此，探索新的生物标志物进行早期诊断以及找到新的治疗靶点仍然是当今肺癌研究的重中之重。癌症被认为是一种与年龄有关的疾病，许多癌症的发病率随着年龄的增长而增加，Klotho基因作为一种与抗衰老有关的抑癌基因越来越多地被发现在人类恶性肿瘤中起着不可忽视的作用。在很多恶性肿瘤如结肠癌、神经胶质瘤、肾癌、肝癌、乳腺癌、宫颈癌中，其编码蛋白Klotho蛋白都是有效的肿瘤抑制因子，尤其在肺癌方面得到了较多的研究和关注^[2⇓⇓⇓⇓-7]^。现总结Klotho（Klotho基因或Klotho蛋白）在肺癌的发病机制和治疗预后中的研究进展，综述如下。

## 1 Klotho基因及相应蛋白概述

Klotho基因家族包括Klotho基因（也称为α-Klotho基因）、β-Klotho基因以及Klotho乳糖根皮苷水解酶（Klotho-lactase-phlorizin hydrolase, Klph）基因。

### 1.1 Klotho基因的结构及其编码蛋白特点

Klotho基因是最早报道的Klotho家族基因，以希腊神话中掌管生命的女神命名，1997年其首次被Kuro等^[8]^在研究自发性高血压时发现。最初，该发现证明了Klotho基因在衰老中起着重要的作用，是一种抗衰老基因。随后，研究^[8]^将Klotho基因缺乏或突变与许多和年龄相关的疾病联系起来，包括心血管、肾脏、肌肉骨骼和神经退行性疾病。人类Klotho基因由5个外显子和4个内含子组成，分布在13q12染色体上，长度超过50 kb^[9]^。

Klotho基因编码了包含1,012个氨基酸的135 kDa大小的I型跨膜蛋白，称为Klotho蛋白（也称为α-Klotho蛋白）^[10]^。它由以下几个结构域构成：（1）胞外氨基端的一个信号序列；（2）两个胞外糖苷酶域（KL1和KL2）；（3）一个跨膜螺旋；（4）胞内羧基端一个含有10个氨基酸的短胞质结构域。循环中的Klotho蛋白包括可溶型和分泌型，前者是由膜蛋白酶裂解全长的Klotho蛋白而产生的^[11]^；后者则是由Klotho mRNA的选择性剪接产生的^[12]^。Klotho蛋白最初在小鼠肾远曲小管中被发现，此后，科学家通过靶向蛋白质组学分析等方法发现Klotho蛋白在人类的多种组织中亦有表达，除表达量最高的肾小管上皮细胞外，包括动脉、上皮、内分泌、生殖和神经元组织中Klotho蛋白均有不同程度的表达^[13]^。

### 1.2 β-Klotho基因的结构及其编码蛋白特点

β-Klotho基因是后来发现的Klotho基因家族成员，尽管β-Klotho基因序列与Klotho基因序列具有高度的相似性^[14]^，但他们却位于不同的染色体上。Klotho基因位于13号染色体上，而β-Klotho基因位于4号染色体上。除此以外，定位于细胞膜的β-Klotho基因编码的β-Klotho蛋白不存在分泌型，该蛋白主要在肝脏和白色脂肪组织中表达^[15]^，且其功能也异于Klotho蛋白。β-Klotho蛋白主要与成纤维细胞生长因子受体1c（fibroblast growth factor receptor 1c, FGFR1c）和FGFR4形成复合物，从而激活下游信号通路，其主要生物学功能包括胆汁酸合成、葡萄糖摄取和脂肪酸代谢^[16]^。

### 1.3 Klph基因及其编码蛋白特点

由于氨基酸结构的相似性Klph基因也被归于Klotho基因家族。基因编码一个单跨膜蛋白Klph，该蛋白主要在肾脏、脂肪组织和眼中表达^[17]^。

本文将重点介绍Klotho在肺癌中的作用，并讨论其在预防和治疗肺癌方面的潜力。

## 2 Klotho抑制肺癌的分子机制

肺癌的发生发展包括一系列的过程如肿瘤相关信号通路的激活、癌细胞的增殖、抗凋亡、迁移、侵袭以及上皮-间质转化（epithelial-mesenchymal transition, EMT）等。因此，对上述肺癌相关分子过程中任何一个环节的影响都可能逆转肺癌发生或延缓肺癌进展。Klotho对肺癌的抑制效应内在分子机制主要体现在以下几点。

### 2.1 Klotho与肺癌相关信号通路的关系

对人肺癌细胞系H1693的研究^[18]^发现，可溶性Klotho蛋白（KL-S）能够通过抑制PI3K相关信号通路驱动的Orai1通道的囊泡胞吐来下调钙离子内流（store-operated calcium entry, SOCE），进一步有助于抑制SOCE介导的肿瘤细胞的迁移。2010年，有学者^[19]^研究发现，Klotho蛋白的过表达能够减少胰岛素样生长因子1（insulin-like growth factor 1, IGF1）/胰岛素诱导的IGF1受体（IGF1 receptor, IGF1R）和胰岛素受体（insulin receptor, IR）的磷酸化，达到抑制肺癌细胞株A549增殖的作用。另外，Chen等^[20]^对A549细胞的研究发现，Klotho蛋白能够通过抑制细胞内的Wnt-TCF/β信号通路连环蛋白的激活来达到抑癌效应，该研究还发现Klotho mRNA剪接变体编码的假体分泌KL-S也有一定程度的肿瘤抑制活性。近些年，有研究^[21]^发现Rab8作为一个与Klotho蛋白相互作用的蛋白，与Klotho蛋白一起在人非小细胞肺癌（non-small cell lung cancer, NSCLC）细胞株表面共表达（共定位），Rab8蛋白能够调节Klotho蛋白在细胞表面表达的水平，且Rab8蛋白过表达可抑制细胞内Wnt信号通路，从而加强了Klotho蛋白对NSCLC细胞的增殖、迁移、侵袭及EMT的抑制作用。该研究在裸鼠的体内实验发现，Klotho基因与Rab8基因共转染的A549细胞在裸鼠体内成瘤速度明显慢于仅转染Klotho基因或转染空载体的A549细胞，且在移植3周后该组肿瘤体积和重量相对更小。对裸鼠肿瘤组织的免疫组化发现，Klotho基因与Rab8基因共转染组的肿瘤组织表面Klotho蛋白表达水平明显高于仅转染Klotho基因组，这些结果表明Rab8蛋白可以在体内增加Klotho蛋白的表达水平，进而增加其抗肿瘤的效应。

### 2.2 Klotho与肺癌细胞凋亡的关系

Klotho蛋白能够通过各种机制增加多种癌细胞的凋亡，如甲状腺癌细胞、结肠癌细胞^[22,23]^，特别是肺癌细胞。有研究^[20]^发现，通过基因转染技术，过表达Klotho蛋白或KL-S的肺癌A549细胞与对照组细胞相比，凋亡率明显增加。另有研究^[24]^揭示，microRNA-10b（miR-10b）与Klotho蛋白表达之间存在显著的负相关关系，转染了miR-10b的肺癌A549细胞和95-D细胞，其Klotho蛋白表达明显降低；而在上述两种细胞中沉默了miR-10b之后，Klotho蛋白表达明显被上调，进一步诱导了细胞凋亡。说明肺癌细胞中Klotho蛋白可能是miR-10b调控的直接靶点，对Klotho蛋白的上调导致了肺癌细胞凋亡的增加。继而，有学者^[19]^深入研究发现Klotho蛋白促使A549细胞快速凋亡的内在机制为：Klotho基因表达上调诱导促凋亡相关基因bax表达上调及抗凋亡基因bcl-2表达下调，进而影响这两种凋亡相关基因的蛋白水平，促进凋亡。随后，又有学者^[25]^发现，Klotho蛋白和可溶性CD40配体（soluble CD40 ligand, sCD40L）结合可以进一步诱导bax蛋白表达增加及bcl-2蛋白表达下降，更大程度地发挥对A549细胞的抗增殖和促凋亡的作用。

### 2.3 Klotho与肺癌细胞EMT的关系

EMT被认为在癌细胞转移/侵袭中扮演了重要的角色，随着EMT的发展，上皮细胞失去了细胞间黏附特性和细胞极性，呈现出高运动间充质细胞的特性^[26]^。有研究^[27]^显示，衰老的肺成纤维细胞（senescent lung fibroblasts, SLF）可大量释放产生白介素6（interleukin 6, IL-6）和IL-8，用含有SLF的条件培养基培养的肺癌细胞株A549，其生存能力和迁移能力明显升高，与EMT相关的标志物α-SMA蛋白表达升高以及上皮细胞钙黏蛋白（epithelial cadherin, E-cadherin）表达下降，且与癌症相关成纤维细胞形成有关的磷酸化信号传导子及转录激活子3（phosphorylated signal tranducers and activators of transcription, p-STAT3）蛋白表达升高和具有抑癌效应的P53蛋白表达下降^[28]^。而过表达Klotho蛋白的SLF则能逆转这种情况，提示Klotho蛋白调节抑制SLF释放IL-6和IL-8，促进肺癌细胞株的P53蛋白表达，并抑制其STAT3蛋白的磷酸化及肺癌细胞EMT，从而间接地抑制了肺癌细胞株的生长和迁移。

综上，Klotho通过抑制肺癌相关信号通路的激活，诱导促凋亡相关蛋白的上调和抗凋亡相关蛋白的下调，增加了抑制肺癌细胞EMT的蛋白表达，抑制了肺癌细胞的增殖、迁移、侵袭及EMT，并促进了肺癌细胞凋亡，这些机制共同构成了Klotho对肺癌抑制作用的分子基础。

## 3 Klotho与肺癌发生发展及预后的关系

之前对Klotho蛋白抑癌分子机制的探讨主要集中在肺腺癌细胞中。临床上，报道主要集中在Klotho蛋白与非腺癌之间的关系上，针对Klotho蛋白与肺癌发生发展和对肺癌患者预后影响的相关报道亦不断涌现。

由于Klotho蛋白在体外实验中对肺癌细胞起负性调控作用的各种证据不断被发现，有理由相信，血清Klotho蛋白水平可能成为癌症的临床生物标志物。Qiao等^[29]^曾报道，血清Klotho蛋白浓度与总癌发生风险呈负相关，然而一项对肺癌患者（包括小细胞肺癌、鳞癌以及腺癌）与正常人血中Klotho蛋白水平的研究^[30]^却显示，两者差异并没有统计学意义。研究^[31]^发现吸烟者和非吸烟者血清中Klotho蛋白水平差异明显，吸烟者Klotho水平明显低于不吸烟者。众所周知，吸烟是肺癌发生的独立危险因素之一，该研究提示Klotho蛋白可能与吸烟导致肺癌发生有一定的关系。

在各种肿瘤中，Klotho蛋白在癌组织中的表达普遍低于癌旁组织。研究^[32]^显示，降低Klotho蛋白表达最常见的机制是通过Klotho基因启动子的甲基化而导致的表观遗传学沉默，且这种启动子的甲基化有随年龄增加而增长的趋势。研究^[20]^发现，Klotho蛋白在肺大细胞神经内分泌癌（large cell neuroendocrine carcinoma, LCNEC）组织中的表达明显低于其癌旁组织，证明Klotho蛋白的低表达与肺癌的发生关系密切。

在Klotho蛋白对肺癌患者疾病的发展与肿瘤侵袭的影响方面，有研究^[33]^发现，在表达Klotho蛋白的早期中心性肺鳞癌患者的肿瘤组织中，免疫组化染色靠近基底膜的上皮细胞的Klotho蛋白表达水平较低，接着又在30例的侵袭性或晚期肺鳞癌患者的外科手术组织中发现只有13%的患者有Klotho蛋白的表达，说明Klotho蛋白的低表达与肺鳞癌的侵袭密切相关。

在Klotho蛋白与肺癌患者预后的关系方面，早在2011年，有日本学者^[34]^对东京医科大学附属医院行外科手术的肺LCNEC患者的回顾性研究发现，Klotho蛋白的高表达与术后患者的良好生存密切相关。在那些无淋巴结转移或淋巴管浸润的原发肿瘤患者中，Klotho蛋白的这种保护作用尤为明显，且Klotho蛋白已被证实为肺LCNEC术后患者预后的独立保护因素，可作为预示肺LCNEC患者术后良好结局的生物标志物。2019年，波兰学者^[35]^的研究再次证实了Klotho蛋白对肺LCNEC患者预后的保护作用。此外，还有学者^[36]^发现Klotho蛋白的表达与局限性小细胞肺癌患者的术后总生存期（overall survival, OS）显著相关（HR=0.088, 95%CI: 0.019-0.409, P=0.002），其高表达预示着患者术后的良好结局。

这些研究显示，虽然Klotho蛋白水平在肺癌患者血清中没有明显下降，但其在肺LCNEC患者组织中的表达明显减少。在临床上，Klotho蛋白低表达与肺癌（尤其是肺鳞癌）的发展与侵袭密切相关，且在预后方面，其高表达与LCNEC和局限性小细胞肺癌患者术后良好生存密切相关。然而，Klotho蛋白与肺腺癌发生发展与侵袭的相关报道较少，尚需进一步探索。

## 4 Klotho与肺癌治疗的关系

肺癌如今最常用的治疗手段包括化疗、靶向治疗及免疫治疗。虽然近些年靶向治疗已经占据了主导，但是传统的化疗仍有一席之地，而这些化疗方法中尤以含铂类的化疗药顺铂最为经典。然而，肺癌患者在化疗中常出现对化疗药物耐受的情况。早在2013年，就有学者^[37]^证实Klotho蛋白能够通过对PI3K/AKT信号通路的抑制使得NSCLC患者对顺铂更加敏感。提示Klotho蛋白不仅能够作为肺癌患者预后的生物标志物，在预示肺癌患者对化疗药物的敏感性上，也起着非常重要的作用。

细胞自噬是一种新型的程序性细胞死亡，其在肿瘤进展中的作用尚不明确。有学者^[38]^发现，对顺铂耐药的肺癌A549细胞A549/DDP和对多西他赛耐药的肺癌SPC-A-1细胞SPC-A-1/TAX与敏感细胞相比，细胞自噬效应明显增强，而过表达Klotho蛋白的这两种耐药细胞中，与细胞自噬相关的Beclin 1蛋白和LC3-II蛋白水平明显下降，说明Klotho蛋白能够抑制耐药细胞的自噬效应，增加肺癌细胞对化疗药物的敏感性，逆转肺癌细胞的耐药。

培美曲塞是一种结构上含有核心为吡咯嘧啶基团的抗叶酸制剂，通过破坏细胞内叶酸依赖性的正常代谢过程，抑制细胞复制，从而抑制肿瘤的生长，也是肺癌常用的化疗药物之一。研究^[39]^发现Klotho蛋白能够抑制肺癌A549细胞的EMT过程，并可通过诱导脂质运载蛋白2（lipid carrier protein 2, LCN2）的表达上升增加了细胞对培美曲塞的敏感性，预示着Klotho蛋白的表达或可作为个体对培美曲塞治疗敏感性的预测因子。

免疫治疗现今已经成为肿瘤患者特别是肺癌患者的一线治疗方案之一。Liang等^[40]^通过对GEO数据库中多个数据集的生物信息学分析发现，Klotho蛋白在包括肺癌之内的多种肿瘤中，与一些免疫治疗相关的生物标志物的表达关系密切；并进一步发现，患者肿瘤组织中Klotho蛋白高表达与抗程序性死亡受体1（programmed cell death protein 1, PD-1）免疫治疗无应答紧密相关，提示Klotho蛋白能影响肿瘤患者抗PD-1免疫治疗的敏感性。

有研究^[41]^发现从蔬菜和水果中提取的植物色素槲皮素具有很高的抗氧化、抗炎症活性，它能使结肠癌细胞中Klotho蛋白的表达量升高，从而抑制癌细胞生长；另有研究^[42]^表明Klotho蛋白已经可以作为一种药物，用于人结直肠癌的辅助治疗，其应用不影响健康的结直肠细胞，但可诱导癌细胞凋亡。这些都说明间接或直接提高Klotho蛋白水平可以对结肠癌起到有效的抑制作用，但在肺癌中尚没有相关研究，或可成为未来肺癌治疗的进一步可探索方向。

总之，在肺癌患者的化疗和免疫治疗中，Klotho蛋白表达的高低与患者对治疗的敏感性密切相关。

## 5 其他蛋白与Klotho蛋白的相互作用及β-Klotho蛋白与肺癌的关系

Chen等^[43]^发现，过去认为对神经元系统的正常功能起重要作用的支架蛋白SH3和多锚蛋白重复结构域1（SH3 and multiple ankyrin repeat domain 1, SHANK1）是潜在的致癌因素，SHANK1蛋白可以与Klotho蛋白及E3泛素连接酶MDM2结合形成复合物，加强了Klotho蛋白与MDM2的相互作用，促进Klotho蛋白泛素化降解，从而促进了NSCLC细胞的增殖、迁移和侵袭。这提示我们，对更多的与Klotho相关蛋白的探索或可使我们发现新的肺癌相关蛋白。

近年来，对β-Klotho蛋白的研究使我们对Klotho蛋白家族有了更加深刻的认识。也有越来越多的报道揭示了β-Klotho蛋白与肺癌的关系。Zhou等^[44]^发现，与健康对照相比，NSCLC患者血清中的β-Klotho蛋白的水平明显降低，患者化疗或靶向治疗后该蛋白水平明显上升，且β-Klotho蛋白高水平组的NSCLC患者OS和无进展生存期较低水平组患者明显延长；在A549细胞中过表达β-Klotho蛋白能够明显抑制其增殖，造成细胞G_1_期-S期阻滞，并诱导凋亡。提示β-Klotho蛋白与Klotho蛋白一样在肺癌中有显著的抑癌效应。

## 6 结语

Klotho基因作为初始发现的具有抗衰老功能的基因，其编码蛋白Klotho蛋白在肺癌组织中表达下调，且这种组织中的低表达与肿瘤侵袭和肺癌患者的不良预后密切相关，提示Klotho蛋白在肺癌中具有抑癌效应。Klotho抑制肺癌发生发展的分子机制包括：Klotho蛋白过表达能抑制IGF1/IGF1R、PI3K以及Wnt-TCF/β信号通路；Klotho蛋白单独以及和sCD40L结合可诱导bax蛋白上调及bcl-2蛋白下调，增加细胞凋亡；Klotho蛋白过表达能够抑制含SLF培养基中SLF细胞释放IL-6和IL-8，促进肺癌细胞株的P53蛋白表达，抑制细胞EMT发生，且抑制了肺癌细胞株的生长和迁移。在肺癌治疗方面，Klotho蛋白的高表达能够增加肺癌细胞对化疗药的敏感性，其机制主要包括抑制PI3K/AKT信号通路、抑制细胞自噬效应以及诱导LCN2的表达。然而，包括肺癌在内的肿瘤组织中Klotho蛋白高表达却与抗PD-1免疫治疗的无应答紧密相关。其他方面，探索与Klotho相结合的其他蛋白或可引导我们发现新的肺癌相关靶点；最后，Klotho蛋白家族的另一成员β-Klotho蛋白也被发现在肺癌中有一定的抑制效应，然而相关研究较少，尚需更多研究证实。目前，关于Klotho与肺癌关系的研究主要集中在基础研究阶段。临床上，对其研究仅局限于有限几种病理类型的肺癌，而在其他肺癌特别是肺腺癌中研究较少，且Klotho与肺癌的靶向治疗关系方面的相关研究较罕见，未来针对这些方面的研究尚需进一步完善。

**Competing interests**

The authors declare that they have no competing interests.
